# From PLP1 Misfolding to Oligodendrocyte Degeneration: A Proteostasis-Centered Framework for Pelizaeus–Merzbacher Disease

**DOI:** 10.3390/cells15151318

**Published:** 2026-07-23

**Authors:** Tianyi Li, Hao Huang, Xiaobin Li, Runlin Leng, Binbin Liu, Guohua Yang

**Affiliations:** 1 Department of Medical Genetics, School of Basic Medical Science, Wuhan University, Wuhan 430071, China; 2 Hubei Province Key Laboratory of Occupational Hazard Identification and Control, Wuhan University of Science and Technology, Wuhan 430065, China

**Keywords:** Pelizaeus–Merzbacher disease, oligodendrocyte, PLP1, unfolded protein response, proteostasis, ERO1α

## Abstract

**Highlights:**

**What are the main findings?**

- PLP1 misfolding initiates a cascade of proteostasis defects involving ER retention, aberrant oligomerization, and dysregulated protein degradation pathways.
- Chronic ER stress, maladaptive UPR activation, and redox homeostasis collapse contribute directly to oligodendrocyte dysfunction and myelin loss in PMD.

**What are the implications of the main findings?**

- A proteostasis-centered model links molecular defects in mutant PLP1 to cellular pathology and clinical heterogeneity across PMD phenotypes.
- Restoring proteostasis and ER homeostasis may provide disease-modifying therapeutic opportunities for PMD and related leukodystrophies.

**Abstract:**

Oligodendrocytes (OLs) are the myelinating cells of the central nervous system (CNS). The *PLP1* gene, predominantly expressed in OLs, encodes proteolipid protein (PLP), a major structural component of CNS myelin that also regulates oligodendrocyte precursor cell (OPC) proliferation, differentiation, and maturation. Pelizaeus–Merzbacher disease (PMD) is a rare X-linked leukodystrophy caused by PLP1 mutations and characterized by defective myelination. Clinical manifestations range from severe connatal PMD to classic PMD and the milder spastic paraplegia type 2 (SPG2), reflecting substantial phenotypic heterogeneity. Beyond disrupting myelin structure, PLP1 mutations impair oligodendrocyte development and function. Increasing evidence indicates that PMD is fundamentally a proteostasis disorder, in which misfolded PLP accumulates within the endoplasmic reticulum (ER), overwhelms ER quality control mechanisms, and triggers chronic unfolded protein response (UPR) activation. Persistent ER stress and maladaptive UPR signaling ultimately promote oligodendrocyte dysfunction and degeneration. Using PMD as a representative model, this review summarizes the relationships between PLP1 mutations and disease phenotypes and discusses the cellular mechanisms by which ER stress and UPR signaling contribute to oligodendrocyte pathology.

## 1. Introduction

In 1885, the German physician Friedrich Pelizaeus first described a familial disorder mainly characterized by nystagmus, developmental delay, spastic paraplegia, and ataxia [1]. Subsequently, in 1910, Ludwig Merzbacher confirmed the X-linked recessive inheritance pattern of the disease [2], which was later named Pelizaeus–Merzbacher disease. 

In this review, we first summarize the molecular regulators governing PLP biosynthesis, trafficking, and myelin assembly, then discuss how distinct PLP1 mutations disrupt ER proteostasis and activate maladaptive UPR signaling, ultimately leading to oligodendrocyte (OL) dysfunction and demyelination. Finally, we highlight emerging therapeutic strategies targeting proteostasis restoration and ER stress modulation.

### 1.1. Pathological Characteristics of PMD

The neuropathological features of PMD are primarily characterized by widespread hypomyelination and white matter atrophy within the central nervous system, accompanied by astrocytic proliferation and subcortical axonal degeneration, with surviving axons frequently showing spherical swelling [3]. Macroscopically, cerebral atrophy is most evident as thinning of the corpus callosum, although the telencephalon and cerebellum are also commonly affected. Microscopically, neuronal loss is observed in multiple brain regions, particularly in the cerebellum, with frequent involvement of the hippocampus, substantia nigra-striatum, and thalamus. Together, the affected white matter contains morphologically heterogeneous “myelin islands,” the distribution of which correlates with disease severity: patients with severe forms exhibit an almost complete absence of white matter myelination, whereas those with milder forms display characteristic “tigroid-like” changes in the central white matter [3]. Histochemical staining further demonstrates abundant small neutral lipid droplets dispersed throughout the astrocytic cytoplasm, suggesting that PMD may be associated with abnormalities in myelin lipoproteins or related lipid metabolism. In the spinal cord, pathological involvement is relatively mild in the thalamocerebellar and anterior spinocerebellar tracts, whereas marked myelin abnormalities are observed in the corticospinal, dorsal, dorsolateral, and dorsal spinocerebellar tracts [4].

### 1.2. Phenotype of PMD: Clinical Characteristics

The clinical onset and manifestations of PMD vary considerably depending on genotype. Most patients present with nystagmus, motor dysfunction (typically progressing from hypotonia to spastic paraplegia), ataxia, dystonia, and cognitive impairment. Patients with severe motor dysfunction-associated respiratory complications may die during early childhood. Connatal PMD represents the most severe form of the disease and is typically fatal before 20 years of age, whereas classic PMD is relatively milder, with patients often surviving into the third to seventh decades of life. Mild forms of PMD commonly manifest as spastic paraplegia type 2 (SPG2) or hypomyelination of early myelinating structures (HEMS).

The clinical spectrum of PMD is highly heterogeneous, encompassing ocular nystagmus, hypotonia, psychomotor retardation (commonly accompanied by intellectual disability), ataxia, epilepsy, and spastic paraplegia arising during infancy or early childhood [5]. Marked phenotypic variability can occur even among affected individuals within the same family. Neurological symptoms usually follow a characteristic pattern of progression: nystagmus commonly develops within the first year of life, and some patients subsequently develop laryngeal stridor and epilepsy as the nervous system matures [6]. Disease severity varies widely, ranging from fatal infantile forms to slowly progressive phenotypes that do not significantly reduce lifespan. Psychomotor retardation generally appears before 2 years of age, with motor impairment typically more severe than cognitive dysfunction. Dystonia and ataxia gradually emerge between 6 months and 2 years of age, hyperreflexia usually develops after 1 year, and the spastic phase typically occurs between 2 and 4 years of age, while ataxic symptoms may persist until approximately 8 years of age [7]. Notably, recent studies have demonstrated that PMD may also involve the peripheral nervous system, characterized by reduced or absent tendon reflexes, abnormal nerve conduction velocities, marked muscle atrophy, and cold extremities, thereby challenging the traditional view that PMD is restricted to the central nervous system [8]. Together, optic atrophy is a common but heterogeneous complication that is typically diagnosed around 6 years of age. Electrophysiological examinations often reveal low-amplitude visual evoked potentials (VEPs) with prolonged latency and waveform distortion [9], and some patients also exhibit abnormal auditory brainstem responses (ABRs) and vestibular dysfunction [10].

As a representative hereditary leukodystrophy (HLD), PMD is characterized radiologically by diffuse hyperintensity of the cerebral white matter on T2-weighted magnetic resonance imaging (MRI-T2WI), with minimal involvement of gray matter, reflecting impaired myelination [11,12,13].

**Figure 1:**
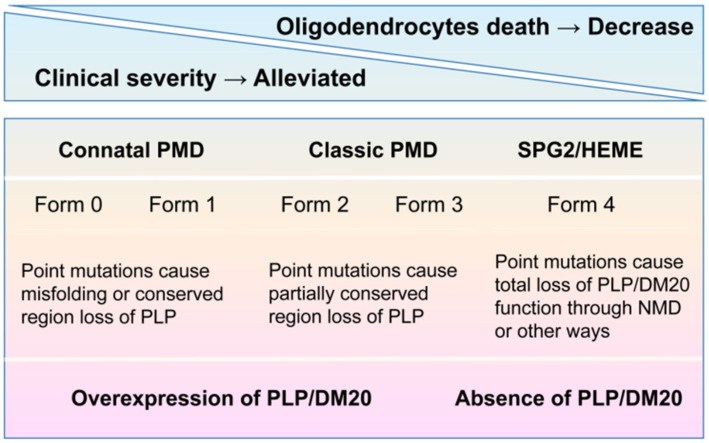
Different phenotypic classifications within the Pelizaeus–Merzbacher disease (PMD) spectrum, the corresponding continuous classification system, and the associated PLP mutation types underlying each subtype. Form 0 represents the most severe phenotype, in which patients completely lose motor function throughout life. Form 1 patients retain only head control, Form 2 patients achieve independent sitting, Form 3 patients are able to stand with support, and Form 4 corresponds to the mildest subtype, Spastic paraplegia type 2 (SPG2), in which patients retain independent ambulation.

Cailloux and colleagues established a continuous classification system for PLP1-related disorders (Forms 0–4), which follows a strict hierarchical progression [7] (Figure 1).

## 2. Molecular Determinants of PMD Pathogenesis

### 2.1. Molecular Regulators of Myelin Assembly and Stability

#### 2.1.1. Role of PLP and DM20

The *PLP1* gene is transcribed in the nucleus and gives rise to the major protein product PLP and its alternatively spliced isoform, DM20. DM20 is expressed before the onset of myelination during early embryonic development, predominantly in oligodendrocyte precursor cells (OPCs), where it serves as an early marker of the OL lineage and contributes to neuronal migration and glial differentiation in addition to myelin formation [14].

Around birth, DM20 progressively accumulates in the myelin sheath, whereas PLP is initially retained within late endosomes/lysosomes of immature OLs. As OLs mature, PLP is rapidly transported from late endosomes/lysosomes to the plasma membrane, accompanied by decreased endocytosis and enhanced exocytosis [15]. During the perinatal period and active myelination, DM20 expression remains relatively stable, whereas PLP expression increases markedly and eventually exceeds that of DM20, becoming the predominant protein in mature myelin. The DM20/PLP ratio is tightly regulated throughout development. Following completion of myelination, PLP continues to be expressed in mature OLs to maintain myelin integrity. During development, DM20 is also expressed in Schwann cells of the peripheral nervous system.

The splice ratio switch from DM20 to PLP during the perinatal period is coordinated by the OL maturation program and developmentally regulated alternative splicing of PLP1 pre-mRNA. The developmental decline in the expression of the trans-acting splicing factors, hnRNP H/F, drives the developmental switch from DM20 to PLP by reducing DM20 splicing isoform while promoting PLP splicing isoform [16,17].

The primary role of PLP1 in myelin assembly and stabilization is to mediate adhesion between the extracellular surfaces of adjacent myelin membranes via its extracellular loops, thereby stabilizing the intraperiod line and compaction of the myelin layers [18]. A secondary role is the metabolic protection of axons, as loss of PLP1 leads to progressive axonal swelling and degeneration [19]. Various classes of PLP1 mutations cause OL dysfunction and death of varying severity, thereby blocking myelin assembly. In contrast, the primary role of DM20 is to govern OL maturation [20]. Expression of a PLP transgene alone cannot achieve PLP incorporation into the myelin membrane in jimpy mice (discussed in the next subsection); partial myelination requires the combined expression of both PLP and DM20 [21]. DM20 can support basic myelin compaction and IPL assembly, but lacks the cholesterol-binding, mitochondrial metabolic regulation, and long-term axonal support capacities of PLP [22].

Within central nervous system (CNS) myelin, PLP/DM20 is the most abundant protein component, followed by myelin basic protein (MBP), 2′,3′-Cyclic Nucleotide 3′-Phosphodiesterase (CNPase), and Oligodendrocyte-Specific Protein (OSP)/Claudin-11. OSP/Claudin-11 and PLP play critical and partially redundant structural and functional roles in maintaining CNS myelin architecture. In single-knockout models, expression of the complementary protein is upregulated (PLP by 48% and OSP by 43%), partially compensating for the deficiency and resulting in relatively mild phenotypes. In contrast, double-knockout mice exhibit impaired myelin compaction, severe disruption of radial component tight junctions and axon–myelin interactions, absence of normally myelinated axons, and a marked reduction in axonal diameter, indicating that these findings highlight the importance of proper myelin compaction in axonal development and long-term maintenance of axonal caliber [23]. These findings may explain why female heterozygous carriers often display mild or even asymptomatic phenotypes.

#### 2.1.2. Other Essential Factors in Myelination

During myelination, both Sirtuin 2 (SIRT2) and Quaking (QKI) play essential regulatory roles, where QKI maintains the stability of MBP mRNA in the cytoplasm and promotes its localization to the myelin sheath by binding to its 3′ untranslated region (3′UTR), and functional deficiency of QKI results in a marked reduction in MBP mRNA expression [24]. SIRT2 is predominantly localized to the noncompact portions of the myelin sheath and the processes of oligodendrocytes, particularly at the juxtanodal and paranodal terminal loops. By deacetylating α-tubulin, SIRT2 negatively regulates OL differentiation and acts as a molecular “brake” during myelination [25]. Interestingly, QKI also indirectly regulates SIRT2 protein abundance and its transport to the myelin sheath through PLP. Loss of QKI6 results in a marked reduction in PLP mRNA, whereas DM20 expression remains largely unchanged. But, before myelination, the absence of PLP does not affect SIRT2 expression or the morphological differentiation of oligodendrocytes, indicating that PLP primarily regulates SIRT2 in mature myelin rather than during the early stages of OL development [26].

Sema6A acts as a positive regulator of OL differentiation and myelination through intracellular signaling mediated by its cytoplasmic domain. Sema6A-deficient mice exhibit significant delays in OL differentiation both in vivo and in vitro, characterized by reduced numbers of PLP, Myelin-Associated Glycoprotein (MAG), and MBP-positive cells and impaired maturation, although these defects gradually recover after adulthood, suggesting the existence of compensatory mechanisms. In addition, in cuprizone-induced demyelination models, Sema6A expression is markedly upregulated in Olig2-positive OL lineage cells within lesioned regions, further supporting its role in promoting OPC differentiation into mature OLs, enhancing process extension and axonal ensheathment, and facilitating endogenous remyelination [27].

Myelin and lymphocyte protein (MAL), a member of the MAL protein family, is a highly hydrophobic integral membrane protein containing four transmembrane domains. MAL primarily regulates the membrane distribution and lateral organization of PLP within specific membrane microdomains [28]. MAL functions to stabilize PLP within specialized membrane microdomains during late myelination and myelin maintenance, thereby facilitating the incorporation of PLP into the expanding myelin sheath independently of complex vesicular transport pathways. Notably, MAL deficiency also gives rise to pathological features reminiscent of PMD [29].

Collectively, these regulators coordinate multiple stages of OL maturation, membrane trafficking, and myelin stabilization. These observations suggest that PLP-associated leukodystrophies are characterized structurally by impaired compact myelin formation and, at the cellular level, by widespread disruption of oligodendroglial homeostasis, which contributes to defective myelin assembly and compaction.

### 2.2. Genotype–Phenotype Correlations in PMD

In the early stages of PMD research, several mouse models with severe congenital phenotypes were identified through extensive investigation, including the point mutation models jimpy and jimpy-msd (hereafter referred to as msd) [30,31]. Subsequent studies revealed that jimpy and msd mutations represent distinct mutation types; nevertheless, both result in accelerated OL death, profound loss of mature oligodendrocytes, and severe hypomyelination [32,33]. Consistently, another point mutation model, SPG-2, corresponding to the jimpy-rsh (rsh) mouse model, exhibits a comparatively milder phenotype [34,35].

The mutation spectrum of the *PLP1* gene includes gene duplications, point mutations, and small insertions or deletions. Epidemiological studies indicate that gene duplications are the most common mutation type, accounting for approximately 60–70% of cases. The size of the PLP1 duplication fragment is closely associated with disease severity in PMD patients, as dose-dependent PLP overexpression disrupts intracellular trafficking and lipid raft assembly, ultimately leading to OL dysfunction [36]. Other dose-sensitive genes located within the duplicated regions have also been implicated in disease pathogenesis [37]. However, fivefold duplications do not produce more severe phenotypes than threefold duplications, suggesting the existence of an upper dosage threshold [38]. Point mutations are the second most common mutation type, accounting for 25–30% of cases. These mutations include missense, nonsense, frameshift, splice-site, and intronic mutations, and are associated with a broad phenotypic spectrum ranging from the relatively mild SPG2 form to the most severe congenital phenotype [39]. Small insertions or deletions are relatively uncommon, accounting for less than 5% of cases. Notably, patients carrying certain loss-of-function mutations, including nonsense, frameshift, splice-site, or deletion mutations, often present with relatively mild SPG2 phenotypes [40,41].

Splice-site mutations or mutations in splicing regulatory elements that alter the PLP/DM20 expression ratio can also lead to disease phenotypes of varying severity [42]. Consistently, mutations in PLP1 exon 3B and intron 3 disrupt exonic splicing silencers (ESSs), alter RNA secondary structures, and interfere with long-distance intronic interactions (LDIs). These abnormalities markedly reduce the PLP1/DM20 ratio and ultimately result in the HEMS phenotype [43]. The PLP-ISEdel (intronic splicing enhancer deletion) mutant model is generated through deletion of an intronic splicing enhancer (ISE) sequence within intron 3 of the *PLP1* gene, resulting in reversal of the normal splice isoform ratio, characterized by reduced PLP expression and increased DM20 expression. This model recapitulates the genetic alterations associated with mild PMD in humans. Mutant mice exhibit impaired behavioral performance as early as 2 months of age, although no significant deterioration is observed between 2 and 4 months. However, after 4 months of age, axonal transport deficits progressively worsen and are accompanied by marked activation of astrocytes (GFAP) and microglia (IBA1, CD68, and MHCII), as well as progressive gliosis. These findings suggest that neuroinflammation contributes to PMD pathology independently of oligodendroglial myelin defects and may therefore represent a therapeutic target [44].

OL apoptosis contributes in severe forms, but hypomyelination and OL death are partially uncoupled—they are separable pathological processes. Firstly, severe PMD contributes to OL apoptosis, OL cell death is increased 2-to 3-fold and occurs by apoptosis in msd mice. Critically, at the time OLs die, they have already differentiated, extended processes that contact axons, and are expressing myelin structural proteins—meaning the cells are mature enough to myelinate but die before completing myelination [45,46]. Secondly, mild PMD is manifested as hypomyelination occurs without abnormal apoptosis. the hallmark of mild disease is hypomyelination whereby OLs differentiate normally but are inefficient at synthesizing myelin sheaths of appropriate thickness [46,47]. Surprisingly, increasing OL survival did not restore subsequent myelination, revealing a second pathological phase—a myelination failure phase that is relatively independent of OL survival [48]. This occurs because natural myelination requires not only the survival of oligodendrocytes but also the proper expression and function of PLP and DM20.

Importantly, identical mutations can produce markedly different phenotypes across distinct genetic backgrounds, highlighting the critical influence of genetic background on disease severity [49].

## 3. Biosynthesis and Functions of PLP

### 3.1. Modification and Processing of PLP

**Figure 2:**
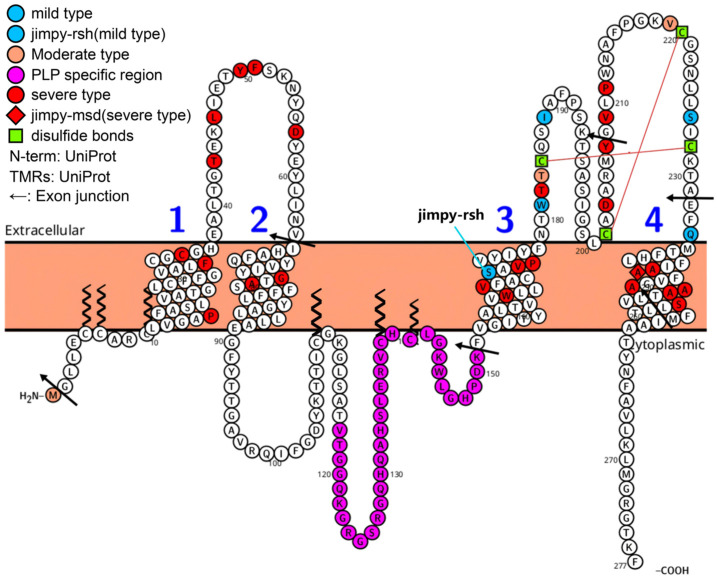
Distribution of PLP1 mutations associated with PMD along the PLP (Uniprot ID: P60201) protein structure. The schematic illustrates the topology of human proteolipid protein 1 (PLP1), a major constituent of central nervous system myelin, highlighting the locations of disease-associated mutations. PLP1 contains four transmembrane domains (TMR1–TMR4, labeled 1–4), extracellular and cytoplasmic loops, as well as a PLP-specific intracellular region shown in purple. Mutations are classified according to clinical severity: mild (blue), moderate (orange), severe (red). Conserved disulfide bonds are marked in green and combined with red solid lines, and exon junctions are denoted by arrows. The black zigzag lines embedded within the membrane represent palmitoylation sites of PLP, a post-translational modification important for membrane association and myelin stability. Disease-causing variants are distributed throughout the protein but are particularly enriched within transmembrane segments and extracellular domains, regions critical for proper folding, trafficking, and membrane integration. The clustering of severe mutations within these structurally important regions supports the notion that PLP1 misfolding and proteostasis disruption are key pathogenic mechanisms underlying OL dysfunction and the phenotypic spectrum of PMD.

The *PLP1* gene consists of seven exons, with a coding sequence spanning 834 bp and encoding a 277-amino-acid protein. It is located on the X chromosome at Xq21.1, spans approximately 17 kb, and encodes a myelin protein containing four transmembrane domains. Alternative splicing within exon 3 generates the DM20 isoform through deletion of the latter portion of the exon. As a result, PLP contains an additional highly conserved 35-amino-acid intracellular loop region that is absent in DM20 (Figure 2). Although DM20 represents a minor isoform in mature myelin, it is the predominant transcript during the developmental stages of OPCs and premyelinating oligodendrocytes (pre-OLs).

PLP is a four-transmembrane-domain protein localized within the myelin membrane and is one of the most abundant proteins in the myelin sheath. As an integral membrane protein, PLP is synthesized by ribosomes associated with the ER. The nascent PLP polypeptide is cotranslationally targeted to the rough ER, where it enters the ER lumen during synthesis. Its four highly hydrophobic transmembrane domains are sequentially inserted into the ER membrane, with both the N- and C-termini oriented toward the cytoplasm, whereas the two extracellular loops (EC1 and EC2) face the ER lumen [50,51]. PLP undergoes lipidation (S-palmitoylation) and disulfide bond formation to maintain its structural integrity and functional stability within the myelin membrane [52,53,54] (Figure 2). Unlike many membrane proteins, PLP does not undergo enzymatic glycosylation [55]. However, pathological conditions may induce non-enzymatic glycation of PLP [56]. Palmitoylation and the intrinsic conformation of PLP promote its partitioning into cholesterol- and sphingolipid-rich lipid raft microdomains, a process that is essential for myelin assembly and stability. Both mutant PLP and PLP overexpression disrupt intracellular trafficking and impair the integration of PLP with lipid rafts [40,57,58].

The topological organization of PLP is highly dependent on disulfide bond formation, primarily within the second extracellular loop (EC2). Two major disulfide bond pairs, Cys200–Cys219 and Cys183–Cys227, are typically formed. Cys108 within the cytoplasmic region is particularly important. In addition to undergoing palmitoylation, it also regulates PLP conformation through a conserved structural mechanism [59]. This conformational change exposes Cys108 and promotes intermolecular disulfide bond formation between PLP molecules, thereby facilitating oligomerization [60]. Accordingly, the three free cysteine residues within TM1 are essential for proper PLP assembly, as their mutation results in protein misfolding, ER retention, and loss of conformational epitopes.

Because PLP does not undergo enzymatic glycosylation, the canonical calnexin (CNX)-mediated quality control mechanism, which monitors glycoprotein folding through recognition of monoglucosylated N-glycans, cannot operate [61]. Nevertheless, CNX is capable of recognizing misfolded PLP through a glycan-independent mechanism, forming stable complexes with mutant PLP, whereas its soluble homolog calreticulin shows no interaction with PLP [55]. CNX can recognize unassembled transmembrane domains in multispanning membrane proteins as a misfolded state, and its interaction with mutant PLP substantially delays PLP turnover. Unlike glycoprotein quality control, in which mannose trimming functions as a switch for substrate release and degradation, the interaction between CNX and nonglycosylated proteins appears to be more stable and persistent [55]. This partially explain the ER retention and reduced degradation of misfolded PLP.

Given the complex folding requirements and membrane insertion of PLP, even subtle mutations can significantly increase the burden on ER proteostasis systems, rendering oligodendrocytes particularly vulnerable to misfolded protein accumulation.

These observations place ER quality control failure upstream of UPR activation and suggest that defective proteostasis is a primary driver rather than a secondary consequence of oligodendroglial pathology.

### 3.2. PLP in Myelin Assembly and Maintenance

**Figure 3:**
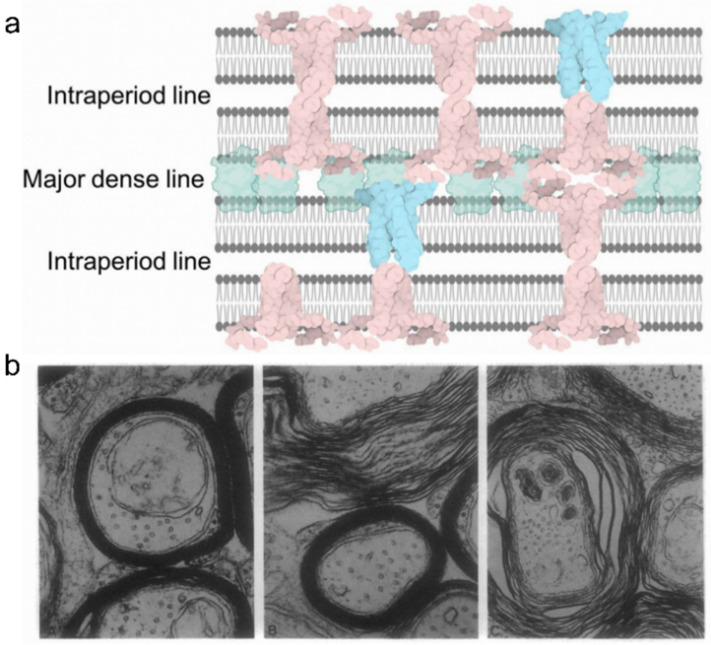
(**a**) Schematic view of CNS compact myelin arrangement. The junctions at the intraperiod line represents those seen in the cryo-EM images. PLP (light pink) and DM20 (cyan) presumably form cis dimers on myelin membranes. MBP (green) forms the major dense line between the cytoplasmic leaflets. Reproduced from Ruskamo et al. 2022 under the terms of the Creative Commons Attribution 4.0 International License. (**b**) Electron micrographs of cross sections of the optic nerve of DM20/neo mice (age 29 days). (A, **left**) Wild-type (XY). Only myelinated axons are visible in this micrograph. The myelin sheaths show a regular compaction visible as major and intraperiod dense lines. (B, **middle**) Heterozygous mutant (XX). Mosaicism of normally myelinating and loosely wrapping OLs due to random X chromosome inactivation is shown. (C, **right**) Hemizygous mutant (XY). Noncompacted voluminously distorted myelin sheaths around large-diameter axons. Small-diameter axons remain unmyelinated. Reproduced from Boison and Stoffel, Proc Natl Acad Sci USA, 1994, 91: 11709–11713. Copyright © 1994 National Academy of Sciences [67,75].

First, OLs undergo extensive plasma membrane specialization to generate a helically extended membrane structure. This structure tightly wraps around axons, forming a multilamellar membrane architecture that functions as an insulating sheath (Figure 3a). Because individual OLs simultaneously myelinate multiple axons, a major challenge during this process is the precise coordination of myelin membrane synthesis and trafficking to maintain membrane domain organization and direct the oriented extension of newly formed myelin sheaths [62,63]. Multiple myelin proteins, including PLP, undergo tightly regulated endocytic sorting and recycling during plasma membrane remodeling [64].

PLP oligomerization is a key determinant of compact myelin formation. First, PLP is stably anchored within cholesterol- and sphingolipid-rich lipid raft microdomains of the OL membrane through palmitoylation-dependent interactions [65]. Second, PLP forms SDS-resistant oligomers, primarily dimers, on the membrane surface. PLP dimers within one membrane layer interact with dimers on the opposing membrane layer through their extracellular domains, thereby forming a transmembrane “zipper-like” structure that tightly apposes adjacent membrane layers (Figure 3a). Loss of both PLP and DM20 results in disappearance of the intraperiod dense line [66,67]. The primary role of PLP in central nervous system (CNS) myelin is structural stabilization rather than myelin assembly, as oligodendrocytes (OLs) lacking both PLP and DM20 are still capable of normal differentiation, survival, and myelin sheath formation [68]. However, the myelin intraperiod line (IPL) exhibits abnormal compaction and markedly reduced mechanical stability, ultimately resulting in late-onset, slowly progressive axonopathy in affected patients often accompanied by demyelinating peripheral neuropathy [18,69]. Notably, patients harboring PLP1 mutations that selectively affect PLP expression while sparing DM20 exhibit entirely normal peripheral nerve function and electrophysiological profiles, indicating that DM20, rather than PLP, is the critical isoform required for maintaining structural and functional integrity in the peripheral nervous system [70].

Formation of the major dense line (MDL) primarily depends on MBP, and axon–OL interactions induce localized translation of MBP [71], which is essential for the initiation of myelin wrapping [72,73]. In contrast, formation of the IPL relies predominantly on PLP. PLP exists mainly as dimers within the membrane, and its extracellular domains (EC1 and EC2) directly mediate adhesion between adjacent membrane layers, thereby promoting tight membrane stacking [50]. By stabilizing the lipid bilayer, PLP maintain the high-resistance and low-capacitance properties of the myelin sheath, which are essential for efficient saltatory conduction of nerve impulses [74,75] (Figure 3b).

These findings highlight that the primary role of PLP extends beyond structural support, and that perturbations in PLP assembly and trafficking can have profound consequences for proteostasis and OL survival.

## 4. Proteostasis Control of Mutant PLP

### 4.1. Oligomerization and ER Retention of Mutated PLP

PLP is synthesized at a relatively rapid rate, becoming detectable within the myelin sheath within approximately 30 min after translation [76]. The ER and secretory pathways of OLs exhibit remarkable sensitivity to cellular stress and perturbation [77]. Mutant PLP, however, is extensively retained within the ER [78], preventing its trafficking to the plasma membrane and ultimately leading to OL apoptosis [45]. Among the underlying pathogenic mechanisms, premature oligomerization of misfolded PLP within the ER is considered a critical factor [79]. Severe phenotypes are generally associated with missense mutations or protein-truncating mutations rather than complete absence of PLP and DM20, as mice lacking both PLP and DM20 survive with normal lifespans and do not exhibit premature death. These findings indicate that severe phenotypes primarily result from toxic gain-of-function effects of misfolded PLP proteins. Mechanistically, in PMD, chronic proteotoxic stress rather than simple loss of PLP1 function is considered the primary driver of oligodendroglial degeneration, as persistent accumulation of misfolded PLP overwhelms ER proteostasis and induces maladaptive UPR activation.

Collectively, the dominant-negative effects of mutant PLP are selective rather than universal. Although mutant PLP does not completely prevent the normal trafficking and localization of wild-type PLP or other myelin proteins, such as MAG and Myelin Oligodendrocyte Glycoprotein (MOG), to the myelin sheath, it does disrupt the normal expression patterns and intracellular trafficking pathways of specific genes and proteins [57,80,81]. Previous studies have demonstrated that the severity of PLP1 mutations positively correlates with the degree of PLP retention within the ER, as well as with the rate and extent of PLP oligomerization [79].

Premature oligomerization induced by mutations within the hydrophobic transmembrane domains (TM1–TM4) is not primarily caused by abnormal disulfide cross-linking within the extracellular domains of mutant PLP, although proper extracellular disulfide bond formation remains essential for correct PLP conformational folding [82]. Instead, the four hydrophobic transmembrane domains of PLP are intrinsically required for normal oligomerization and assembly of the wild-type protein. In native PLP monomers, the TM4 interaction interface is masked by proper protein folding. However, the A242V mutation identified in the msd model disrupts monomer conformation, exposing a high-affinity hydrophobic interaction surface involving Val242. This conformational alteration drives abnormal premature oligomerization of PLP, resulting in ER retention and disease pathogenesis. This mechanism explains how a single amino acid substitution can produce severe neurological disease [83]. Similarly, the TM1–TM3 regions also contribute to normal PLP dimerization, and mutations within these domains lead to aberrant dimer formation [84].

Pathogenic mutations within the EC2 domain of PLP induce abnormal intermolecular cross-linking between unpaired cysteine residues, including Cys200–Cys219, Cys183–Cys227, and Cys108, generating nonfunctional protein dimers that accumulate within the ER and activate the UPR, ultimately leading to OL death [85,86]. These studies further demonstrated that the previously proposed seven-amino-acid peptide sequence within the cytoplasmic loop does not represent the core ER retention signal [87]. The intramolecular disulfide bond between Cys183 and Cys227, located proximal to the membrane, is particularly critical for ER quality control (ERQC), and mutations affecting this region result in severe ER retention. Notably, substitution of Cys200 and Cys219 within EC2-mutant PLP can partially reverse ER retention [82].

In the msd model, mutations involving four key cysteine residues within the EC2 extracellular domain still result in pronounced ER retention and efficient formation of SDS-resistant oligomers, indicating that PLP oligomer stability depends predominantly on non-covalent interactions [79]. More importantly, premature oligomerization of mutant PLP, rather than ER retention alone, appears to be the principal driver of the severe phenotype. Accordingly, polymers generated through premature oligomerization exhibit stronger interactions with ER quality control proteins, including calnexin (CNX), than isolated mutant PLP oligomers [79].

Taken together, these findings indicate that premature oligomerization and ER retention of mutant PLP represent central pathogenic events that initiate proteostasis collapse in oligodendrocytes.

### 4.2. Intracellular Trafficking and Degradation of PLP

#### 4.2.1. Lipid Raft Trafficking

Within the OLs, cholesterol and glycolipids undergo phase separation within the ER and trans-Golgi network (TGN), forming membrane microdomains known as “lipid rafts” through which PLP is incorporated into the plasma membrane [88,89]. Under physiological conditions, misfolded proteins are recognized, refolded, or degraded by the endoplasmic reticulum quality control (ERQC) system, thereby maintaining ER homeostasis. However, PLP mutations or PLP overexpression result in marked ER retention. Newly synthesized PLP entering the ER is immediately associated with molecular chaperones, including BiP, calnexin, and members of the protein disulfide isomerase (PDI) family, which facilitate proper protein folding and disulfide bond formation [55,82,90]. Proteins that fail to fold correctly are retained within the ER by these chaperones, allowing additional opportunities for refolding before correctly folded proteins are transported to the Golgi apparatus (GA) and subsequently delivered to the plasma membrane.

After trafficking to the Golgi apparatus, a portion of PLP is directly sorted into transport vesicles/tubular intermediates destined for recycling endosomes (REs), which function as intermediate sorting stations before fusion with the soma “apical-surface-like” plasma membrane via the VAMP3–Stx4 pathway [91,92]. Proper formation of the “basolateral-surface-like” myelin sheet requires the coordinated action of VAMP2 and VAMP3; deletion of either alone does not impair myelin formation, likely because of compensatory mechanisms, whereas simultaneous loss of both results in severe hypomyelination [93,94]. Another fraction of PLP may be transported directly from the trans-Golgi network (TGN) to the plasma membrane through non-coated vesicles or tubular carriers, bypassing REs [95].

#### 4.2.2. Endosomal Recycling

Following delivery to the plasma membrane, part of the PLP pool is internalized into the endosomal system and trafficked to late endosomes/lysosomes (LE/Lys) through VAMP7–Stx3-dependent pathways, where it is either degraded or stored until signal-dependent release. Redistribution of PLP is regulated by neuronal signaling pathways involving inactivation of soluble Rho-GTPases, and treatment with dibutyryl-cAMP (db-cAMP) mimics neuronal stimulation and promotes PLP translocation to the plasma membrane [15,96,97]. Collectively, the VAMP7–Stx3 pathway is essential for efficient incorporation of PLP into the myelin sheath, as inhibition of Stx3 results in intracellular accumulation of PLP.

Sulfatides, generated through sulfation of galactosylceramide (GalC) by cerebroside sulfotransferase (CST), trigger conformational and microdomain transitions of PLP at the plasma membrane, converting PLP from TX-100-insoluble membrane domains to CHAPS-resistant/insoluble domains that facilitate subsequent endocytosis and trafficking. Sulfatides also alter PLP surface conformation, exposing the O10 epitope. Appearance of the O10 epitope indicates completion of the PLP conformational transition (ET3 → O10), preparing PLP for entry into the transcytotic pathway toward the myelin sheath. Notably, during late brain development, MAL suppresses this conformational transition, thereby permitting PLP to laterally diffuse directly from the plasma membrane into the myelin membrane [28]. In the absence of sulfatides, PLP remains trapped at the cell surface, impairing efficient transport into the myelin sheath and thereby disrupting normal myelin assembly [98]. GalC is synthesized by ceramide galactosyltransferase (CGT) and is highly enriched in OL myelin membranes. GalC deficiency leads to aberrant PLP trafficking and sorting [88].

#### 4.2.3. Lysosomal Degradation and ERAD-Mediated UPS

Wild-type PLP is primarily degraded through the lysosomal pathway, whereas mutant PLP is mainly cleared through proteasome-dependent degradation. More severe mutations are associated with slower degradation kinetics and greater accumulation of mutant PLP within the ER [99]. When protein folding repeatedly fails or is intrinsically defective, misfolded PLP is directed into ER-associated degradation (ERAD) pathway [57,100]. Because PLP lacks enzymatic glycosylation modifications, mutant PLP cannot be efficiently recognized through the classical CNX/CRT cycle or EDEM-mediated pathways [101]. Misfolded PLP is subsequently retrotranslocated across the ER membrane through reverse translocation complexes, such as the Derlin complex or Sec61 channel, and degraded via the ubiquitin–proteasome system (UPS). Certain milder mutations permit normal ER and GA export but lead to excessive mistargeting toward lysosomal degradation, thereby reducing membrane localization of PLP [102]. PLP overexpression caused by duplication mutations results in accumulation of PLP within late endosomes/lysosomes without efficient transport to the plasma membrane [103]. Other mutations, such as W162L, cause abnormal PLP localization around the nuclear envelope (NE), which is associated with relatively mild clinical phenotypes [104].

Interestingly, Rab27b regulates PLP transport by controlling exocytosis of late endosomes/lysosomes toward the plasma membrane and thereby contributes to OL myelination in the CNS [105]. Rab7b, another Rab family GTPase, mediates retrograde transport from late endosomes/lysosomes back to the trans-Golgi network (TGN) [106]. In the msd model, Rab7b knockdown significantly restores cellular morphological differentiation, upregulates OL differentiation markers, and alleviates ER stress, because Rab7b deficiency promotes trafficking of mutant PLP toward late endosomes/lysosomes and accelerates its degradation [107]. These trafficking defects are not merely secondary consequences of PLP misfolding, but actively contribute to proteostasis imbalance by altering the distribution, degradation, and accumulation of PLP within the endomembrane system.

#### 4.2.4. Autophagy and NMD

Cells can eliminate damaged ER fragments together with associated substrate proteins through ER-phagy, whereby ER fragments are engulfed and delivered to lysosomes for degradation, thereby maintaining cellular homeostasis [108,109,110]. However, there is currently no direct experimental evidence that PLP or DM20 is degraded through the ER-phagy pathway. In OLs, macroautophagy degrades myelin proteins including PLP through amphisome-mediated turnover, and its disruption leads to intracellular PLP accumulation and cytotoxicity [111,112]. Consistently, autophagy is dynamically regulated across the OL lineage, serving distinct roles at different developmental stages [113,114,115].

Notably, when mutations occur within non-terminal coding exons and induce frameshifts that generate premature termination codons (PTCs), the mutant transcripts are typically degraded through nonsense-mediated mRNA decay (NMD), often resulting in relatively mild phenotypes [116]. In contrast, mutations occurring within the final exon can evade NMD, leading to a more severe phenotype [117].

Thus, impaired trafficking and degradation pathways further exacerbate proteotoxic stress, creating a vicious cycle of PLP accumulation and ER dysfunction.

### 4.3. Molecular Chaperones in PLP Folding and Quality Control

Because mutant PLP frequently escapes productive folding and accumulates within the ER, molecular chaperone systems become critically important for determining whether mutant proteins undergo refolding, ER retention, or degradation.

Disulfide bond isomerases (PDIs), primarily represented in mammals by ERp57, ERp44, and ERp72, function not only as redox enzymes but also as molecular chaperones [118,119]. These proteins bind partially folded or misfolded substrates through their hydrophobic domains, thereby stabilizing folding intermediates, preventing protein aggregation, and providing an appropriate microenvironment and temporal window for disulfide bond rearrangement [120]. However, when PLP1 mutations—particularly those affecting the EC2 domain—alter the spatial conformation of PLP, aberrant disulfide cross-linking occurs among PLP homologs at residues Cys108, Cys183, Cys200, Cys219, and Cys227, thereby preventing PDI-mediated correction of protein folding.

Many ER-resident molecular chaperones, including PDI and CNX, exhibit activities that are highly dependent on the oxidative environment of the ER. By undergoing conformational changes themselves, these chaperones facilitate the folding of nascent polypeptides and prevent protein misfolding and aggregation [121]. In particular, their functions associated with disulfide bond formation and rearrangement require an appropriate redox potential. Under physiological conditions, the ER maintains a highly oxidative environment, largely due to its elevated concentration of oxidized glutathione (GSSG), which provides the chemical driving force necessary for chaperone-mediated catalytic activity.

BiP (GRP78/HSPA5), the major ER-resident Hsp70 chaperone, recognizes exposed hydrophobic regions within misfolded PLP and prevents aberrant aggregation [122]. Mechanistically, PLP1 mutations alter the amino acid sequence of PLP, leading to aberrant protein conformation and disruption of its native folding state. Consequently, hydrophobic residues that are normally buried within the protein core or embedded in the membrane become exposed. BiP interacts only transiently with newly synthesized wild-type PLP, whereas misfolded msd-PLP forms stable and persistent BiP-associated complexes, thereby promoting prolonged ER retention and unresolved proteostatic stress [55]. This prolonged interaction serves as a critical signal of protein misfolding.

In addition to functioning as a molecular chaperone during ER protein folding, BiP also participates in ERAD. BiP, together with its co-chaperone ERdj5, acts on substrate proteins and interacts with Sel1L in the ER membrane retrotranslocation complex to mediate substrate retrotranslocation [123,124,125]. However, PLP mutations induce premature oligomerization, resulting in recognition and retention by the ER quality control (ERQC) system within the ER. The increased molecular size of oligomerized PLP together with exposure of hydrophobic cores further promotes polymerization, thereby impairing efficient ERAD-mediated clearance and disrupting ER homeostasis. Together, knockout of Sel1L, a key ERAD component, results in progressive thinning of central nervous system (CNS) myelin, motor dysfunction, and premature lethality [126].

## 5. UPR-Mediated Oligodendrocyte Apoptosis in PMD

### 5.1. PLP Misfolding and Activation of the Unfolded Protein Response

**Figure 4:**
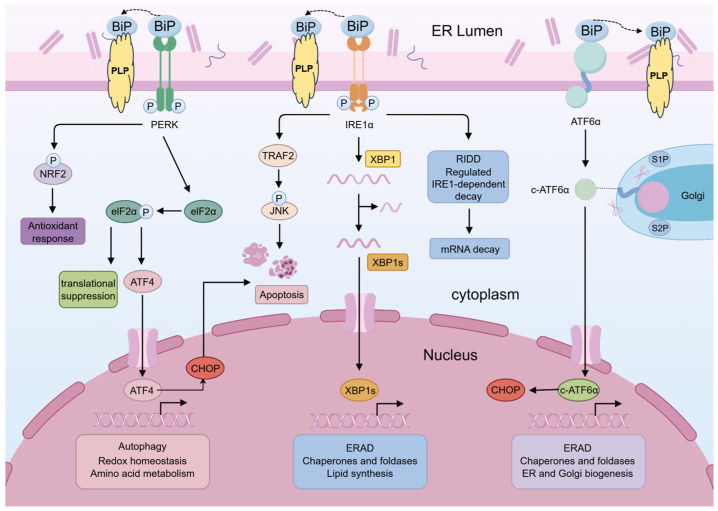
UPR signaling pathways activated during ER stress. Under basal conditions, the ER chaperone BiP binds and suppresses the three principal ER stress sensors—PERK, IRE1α, and ATF6α. Accumulation of misfolded PLP in the ER lumen sequesters BiP, leading to activation of these signaling branches. Activated PERK phosphorylates eIF2α, resulting in transient attenuation of global protein synthesis while selectively promoting ATF4 translation. ATF4 induces genes involved in autophagy, redox homeostasis, amino acid metabolism, and, under sustained stress, the pro-apoptotic transcription factor CHOP. IRE1α mediates unconventional splicing of XBP1 mRNA to generate the active transcription factor XBP1s, which upregulates ERAD, molecular chaperones, foldases, and lipid biosynthesis pathways. IRE1α also activates TRAF2–JNK signaling and promotes regulated IRE1-dependent decay (RIDD) of selected mRNAs. Upon ER stress, ATF6α translocates to the Golgi apparatus, where cleavage by S1P and S2P releases the active fragment c-ATF6α, which enters the nucleus to induce ERAD components, chaperones, and genes involved in ER and Golgi biogenesis. Together, these pathways coordinate adaptive proteostasis responses or trigger apoptosis when ER stress becomes chronic or overwhelming.

When excessive misfolded PLP proteins exceed the processing capacity of ERAD, the UPR is activated. The UPR transiently attenuates global protein synthesis while upregulating chaperone expression to enhance ER repair capacity [127]. Impairment of the ERAD pathway can also trigger ER stress [126]. BiP acts as a key sensor and regulator of ER stress (ERS). Under physiological conditions, BiP binds extensively to the luminal domains of the three ER transmembrane stress sensors—IRE1, PERK, and ATF6—thereby maintaining them in an inactive state. Upon accumulation of misfolded proteins within the ER lumen, BiP dissociates from these stress sensors, leading to activation of the IRE1/PERK/ATF6 signaling pathways and initiation of the UPR [128] (Figure 4). Subsequently, cells initiate adaptive responses or undergo apoptosis depending on the severity and duration of ER stress [129]. The activation profiles of these three UPR branches vary among different cell types and in response to distinct ER stress inducers, such as dithiothreitol (DTT), thapsigargin (Tg, a SERCA Ca^2+^-ATPase inhibitor), and tunicamycin (Tm, an N-glycosylation inhibitor) [130].

Interestingly, under chronic and unresolved ER stress conditions in models of autosomal dominant retinitis pigmentosa (RP), the three branches of UPR do not exhibit synchronized activation. IRE1 signaling declines rapidly (within approximately 8 h), ATF6 signaling subsequently decreases more gradually, whereas PERK signaling remains persistently active. Attenuation of the IRE1 pathway represents a critical event in ER stress-induced cell death. Chemogenetic maintenance of IRE1 signaling markedly improves the survival of HEK293 cells under chronic ER stress, supporting a causal relationship between sustained IRE1 activity and cell fate determination [131]. Consistently, in the jimpy model, PERK knockout enhances the expression of IRE1- and ATF6-dependent target genes, restores proteostasis, and prevents OL apoptosis [132].

Notably, the PERK and ATF6 pathways are essential for maintaining the function and survival of mature OLs and adult myelin, but exert minimal effects on OLs undergoing developmental myelination [111]. Importantly, the UPR exerts a dual role in myelinating disorders: under mild or acute stress conditions, it functions protectively by reducing the burden of protein synthesis, enhancing protein folding capacity, and promoting ERAD, thereby supporting the survival of myelinating cells. In contrast, under prolonged or severe stress, the persistent UPR activation may shift toward pro-apoptotic signaling, ultimately leading to myelinating cell death and demyelination [133].

In PMD, this adaptive response becomes maladaptive due to the persistent presence of misfolded PLP, leading to chronic UPR activation and failure of proteostasis restoration. Meanwhile, UPR activation precedes activation of the innate and/or adaptive immune systems [134].

#### 5.1.1. PERK Signaling in Oligodendrocyte Apoptosis

The PERK pathway plays a critical role in OLs within the central nervous system (CNS). ER stress induces PERK homodimerization and activation, leading to phosphorylation of eIF2α and generation of p-eIF2α. Subsequently, p-eIF2α suppresses global protein translation [135] (Figure 4). Transient inhibition of protein synthesis represents a protective cellular stress response; however, PLP mutations during postnatal development in PMD induce chronic ER stress, ultimately resulting in OL apoptosis and impaired myelination [46,136].

Under conditions of global translational repression, ATF4 mRNA expression is paradoxically increased [137], owing to the presence of two upstream open reading frame (uORF) regulatory elements within the 5′ untranslated region of ATF4 mRNA [138,139]. ATF4 subsequently translocates into the nucleus, where it regulates the transcription of downstream UPR-associated genes, including *CHOP*, *TRIB3*, *GADD34*, and *ATF3*. Although *ATF3* expression is markedly upregulated, *ATF3* deficiency does not reverse the apoptotic phenotype observed in severe OL pathology, indicating that ATF3 is not a critical mediator of apoptosis [140]. GADD34 functions as a negative feedback regulator by promoting dephosphorylation of p-eIF2α, thereby restoring global protein translation [141]. Notably, during normal OL maturation, the IRE1 and ATF6 branches of the UPR are strongly activated during active myelination, likely reflecting the high membrane synthesis burden in OLs. In contrast, the PERK pathway activation is substantially attenuated, presumably to avoid excessive inhibition of global protein translation [137].

Importantly, ATF4-mediated induction of CHOP promotes CHOP binding to the promoter regions of the *ERO1α* and *GADD34* genes, thereby specifically enhancing their transcription. Previous studies have demonstrated that, in mouse embryonic fibroblasts (MEFs) under ER stress, CHOP directly binds to the promoter regions of Ero1α and GADD34, indicating that they are direct transcriptional targets of CHOP [142]. Under ER stress conditions, ATF4 and CHOP also contribute to cell death by transcriptionally activating components of the protein translation machinery, rather than by directly inducing apoptotic genes. Restoration of mutant protein translation exacerbates ER overload, leading to excessive reactive oxygen species (ROS) production and ATP depletion through the ERO1α pathway, thereby inducing oxidative stress and energy exhaustion that ultimately trigger cell death [143].

Studies have shown that mutant PLP in jimpy model mice exhibits strong mitochondrial co-localization, accompanied by elevated levels of ROS, superoxide anion (O_2_^−^), and glutathione (GSH), indicating abnormal mitochondrial redox homeostasis and activity. Together, the jimpy model displays increased expression of peroxisomal markers, including ALDP and ACOX1, significantly elevated basal expression of the *Abcd2* (*ALDRP*) gene, and enlarged rod-shaped mitochondria compared with the smaller and irregular mitochondria observed in wild-type OLs. These morphological abnormalities are associated with mutant PLP expression [144]. Repetitive PLP mutations also exhibit marked mitochondrial localization through the Mia40/Erv1-dependent mitochondrial import pathway, resulting in mitochondrial damage, cytoplasmic release of cytochrome c, and increased PARP cleavage, a hallmark of apoptosis execution. Similarly, mice carrying these repetitive mutations exhibit elevated cytoplasmic cytochrome c levels and increased nuclear AIF accumulation, further supporting a link between mitochondrial dysfunction and apoptosis [145]. Consistently, the cysteine residue and structural features within the PLP-specific 35-amino-acid domain, which is absent in DM20, are essential for mitochondrial targeting; therefore, mutations in this region result in PLP retention in the ER [145].

However, the function of CHOP is not restricted to the promotion of apoptosis. In the rsh model, CHOP deficiency instead markedly exacerbated disease severity and shortened lifespan, suggesting that CHOP may exert anti-apoptotic or pro-survival effects in OLs [127,146]. Nevertheless, in PMD and other experimental disease models, CHOP knockout partially rescues the phenotype, indicating that CHOP possesses both pro-survival and pro-apoptotic functions in OLs. This dual role of CHOP has also been reported across multiple cell types and disease contexts [49,147,148]. Notably, CHOP knockout in severe disease models, including msd and jimpy mice, failed to rescue extensive OL loss or premature mortality [132]. Nevertheless, CHOP upregulation in OL-related disorders is frequently associated with apoptotic progression, whereas in spinal cord injury (SCI), CHOP deficiency significantly attenuates the injury-induced UPR activation and promotes improved recovery outcomes [149].

Although Caspase12 is activated under these conditions, it does not appear to play a direct role in apoptosis, as Caspase12 deficiency fails to reduce cell death, while Caspase9 and Caspase3 remain activated in mutant mice [150]. ATF3 is also induced but is not directly associated with OL apoptosis [148].

The context-dependent role of PERK signaling remains incompletely understood. While transient translational attenuation appears protective during acute ER stress, prolonged PERK activation may exacerbate proteostatic collapse by suppressing myelin protein synthesis and promoting CHOP-dependent apoptosis. The discrepancy among models may reflect differences in mutation severity, developmental stage, and ER stress duration.

Thus, prolonged PERK activation represents a key mechanism by which proteostasis failure is translated into OL dysfunction and apoptosis.

#### 5.1.2. IRE1 and ATF6 Signaling in ER Stress Adaptation

Compared with PERK signaling, IRE1 and ATF6 pathways primarily support adaptive responses; however, their insufficient or transient activation may contribute to the failure to restore proteostasis under chronic stress conditions.

The IRE1α pathway is mediated by IRE1α, which possesses both protein kinase and endoribonuclease (RNase) activities. Under basal conditions, IRE1α remains inactive through its interaction with the ER-resident chaperone BiP via the N-terminal luminal domain (NLD). Upon dissociation of BiP, IRE1α undergoes homodimerization and autophosphorylation, leading to NLD dimerization and subsequent activation of its cytoplasmic kinase and RNase domains [151,152] (Figure 4). Activated IRE1α specifically excises a 26-nucleotide intron from X-box binding protein 1 (XBP1) mRNA, generating the spliced form of XBP1 (XBP1s), which functions as an active transcription factor. Mutations in key amino acid residues within the NLD impair dimerization [153].

Notably, activation of IRE1α is not solely dependent on BiP dissociation induced by unfolded proteins but can also be triggered through its transmembrane domain (TMD) in response to saturated fatty acid palmitoylation [154,155]. Interestingly, the ability of IRE1α to sense ER membrane abnormalities is independent of the specific amino acid sequence of the TMD; rather, the presence of a functional membrane-anchoring domain is sufficient to detect membrane perturbations and initiate activation [156]. However, residues W457 and S450 within the TMD are essential for stabilizing the dimerization interface, and mutations at these sites disrupt IRE1α dimerization [157]. XBP1s plays a pivotal role in upregulating ER membrane-associated proteins and molecular chaperones, thereby enhancing protein folding, secretion, and ERAD-related gene expression. Furthermore, XBP1s alleviates the mRNA burden through regulated RIDD and promotes phospholipid synthesis to expand ER membrane surface area, collectively contributing to the attenuation of ER stress. Nevertheless, sustained activation of the IRE1 pathway can also promote apoptosis [158].

In the ATF6 pathway, activated ATF6α translocates to the GA, where it is cleaved by site-specific proteases to release its cytosolic fragment, ATF6α-p50. This fragment subsequently translocates into the nucleus and induces the expression of molecular chaperones and ERAD-associated components (Figure 4). However, ATF6 expression levels in the msd mouse model show minimal differences compared with wild-type controls, suggesting that the UPR activation in OLs within this model does not efficiently engage the ATF6 branch [89]. Although ATF6α deficiency does not affect normal development, OL survival, myelination, or myelin ultrastructure under physiological conditions, it significantly increases OL susceptibility to cell death and myelin damage under inflammatory stress [159].

Interestingly, previous studies have demonstrated that under ER stress conditions in mouse embryonic fibroblasts (MEFs), ATF6α and XBP1 form heterodimers with greater binding affinity for the unfolded protein response element (UPRE) than XBP1 homodimers [160]. ATF6α and XBP1 also exhibit synergistic interactions, as activated ATF6 enhances XBP1 mRNA transcription following nuclear translocation [161]. 

ATF6α and XBP1 display partial functional redundancy, since ATF6α deficiency does not markedly alter the expression of downstream UPR target genes [162]. Notably, IRE1 signaling undergoes self-amplification through the kinase–JNK pathway, whereas ATF6 appears to function as a negative regulator that restrains excessive and sustained IRE1 activation during later stages of ER stress, thereby preventing signaling dysregulation [163]. Recent studies further revealed that XBP1s induces the expression of molecules such as TMED9, which stabilize ATF6 and prevent its degradation, thereby sustaining the pro-survival signaling mediated by ATF6 during prolonged ER stress [164].

### 5.2. ER Redox Homeostasis and Oxidative Stress

**Figure 5:**
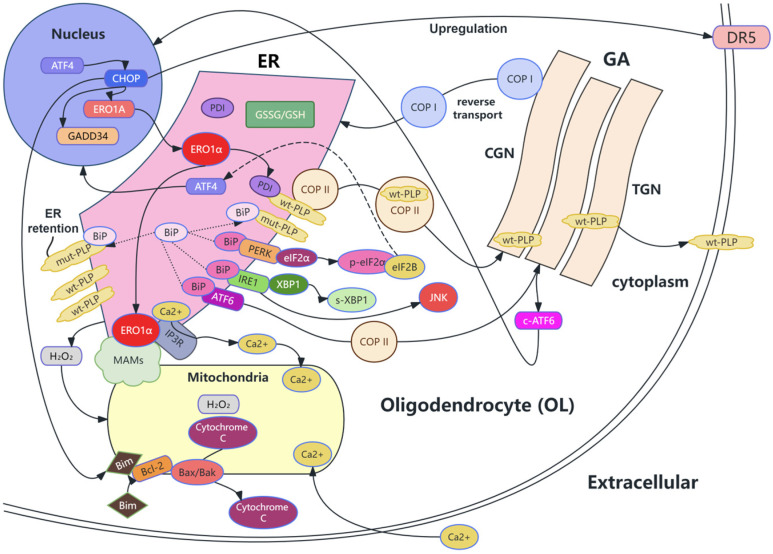
Mechanism linking PLP misfolding, proteostasis disruption, OL degeneration in PMD, and wild-type PLP transportation. Mutant PLP proteins fail to achieve their native conformation and accumulate within the ER of oligodendrocytes, where they are retained by molecular chaperones such as BiP and PDI. Persistent accumulation of misfolded PLP activates the UPR through the PERK, IRE1, and ATF6 pathways, leading to phosphorylation of eIF2α, induction of ATF4, XBP1 splicing, JNK activation, and ATF6 processing. Sustained ER stress promotes CHOP-, ERO1α-, and GADD34-dependent maladaptive signaling, disrupting ER redox homeostasis and enhancing ROS production. Elevated ERO1α activity at mitochondria-associated ER membranes (MAMs) stimulates IP3R-mediated Ca^2+^ release, resulting in mitochondrial Ca^2+^ overload, cytochrome c release, and activation of the intrinsic apoptotic pathway through Bim and Bax/Bak. In parallel, defects in COPII-mediated ER export and COPI-dependent retrograde trafficking impair PLP transport through the Golgi apparatus, further exacerbating proteostasis collapse. Upregulation of death receptor signaling, including DR5, amplifies apoptotic responses. Together, these interconnected pathways drive OL dysfunction, degeneration, and myelin loss in PMD.

msd-PLP induces depletion of key ER molecular chaperones carrying C-terminal KDEL retrieval signals, including PDI, CALR, and GRP78, resulting in their redistribution from the ER to the cytoplasm. Notably, msd-PLP causes ER Ca^2+^ depletion and promotes fragmentation of the GA. PLP mutations also impair COPII vesicle assembly and trafficking at the rough endoplasmic reticulum exit sites (ERES), which may subsequently disrupt GA-to-ER COPI-mediated retrograde transport. The severity of these intracellular pathological alterations, including molecular chaperone depletion, vesicular transport defects, and Golgi fragmentation, differs between mild and severe PLP mutants and correlates with the clinical severity associated with each mutation [165,166] (Figure 5). Restoration of ER calcium homeostasis—for example, through the SERCA activator CDN1163, the IP3R inhibitor 2-APB, or the RyR inhibitor dantrolene—can partially rescue defects in COPII assembly and vesicular transport.

As discussed previously, ER redox homeostasis is essential for proper protein folding, as PDI-mediated folding depends on dynamic conformational changes within substrate proteins [121,167]. These processes are tightly regulated by ER redox-associated molecules and proteins, particularly the GSSG/GSH system and ERO1α [168]. In the cytoplasm, GSH concentrations greatly exceed those of GSSG, with ratios ranging from approximately 30:1 to over 100:1. This highly reducing environment enables GSH to reduce disulfide bonds and maintain protein sulfhydryl (–SH) groups in a reduced state. In contrast, GSSG levels are substantially elevated in the ER, resulting in a markedly lower GSH/GSSG ratio. The relatively oxidizing environment within the ER thermodynamically favors disulfide bond formation [169,170] (Figure 5).

ERO1α directly oxidizes PDI, converting it into its oxidized disulfide form (–S–S–) [171]. During this process, the first cysteine pair within the ERO1α catalytic center is reduced to the thiol form (–SH). Subsequently, the second cysteine pair re-oxidizes the reduced first cysteine pair, restoring it to the disulfide state. Simultaneously, the second cysteine pair transfers two electrons to the flavin adenine dinucleotide (FAD) cofactor bound to ERO1α, generating reduced FADH_2_. FADH_2_ subsequently transfers electrons to molecular oxygen, producing H_2_O_2_ and thereby completing the electron transfer cascade [172].

Oxidized PDI subsequently interacts with newly synthesized substrate proteins and catalyzes disulfide bond formation through thiol–disulfide exchange reactions, during which PDI itself becomes reduced to the thiol state (–SH). Reduced PDI then recognizes and reduces incorrectly paired disulfide bonds within substrate proteins, converting them back into free thiol groups through additional thiol–disulfide exchange reactions, while PDI is re-oxidized to the disulfide state (–S–S–). If incorrect disulfide bonds persist, oxidized PDI repeatedly catalyzes rearrangement reactions until the substrate protein achieves its native disulfide configuration. GSH subsequently reduces oxidized PDI back to its reduced form, allowing continued correction of mispaired disulfide bonds. Each catalytic rearrangement requires the transfer of two electrons from GSH to maintain PDI activity [173]. Persistent production of misfolded PLP proteins drives sustained PDI activity and rapidly depletes ER glutathione (GSH) reserves. Because GSH is a major ER antioxidant, its depletion compromises ROS scavenging capacity and disrupts ER redox homeostasis. As a result, even low levels of ROS cannot be efficiently eliminated, leading to rapid ROS accumulation. Accordingly, depletion of GSH further increases the GSSG/GSH ratio, disrupting ER redox homeostasis.

Importantly, the UPR activated during ER stress is initially intended to enhance protein folding capacity and facilitate degradation of misfolded proteins. However, CHOP upregulation through the PERK pathway also induces increased expression of ERO1α [142]. ERO1α enhances IP3-induced calcium release (IICR) by relieving ERp44-mediated inhibition of IP3R, thereby promoting ER Ca^2+^ depletion and triggering apoptosis [174]. ERO1α localizes to MAMs in a redox-dependent manner and regulates calcium signaling between the ER and mitochondria. A reducing environment facilitates its dissociation from MAMs into the secretory pathway [175] (Figure 5). Excessive cytosolic calcium subsequently activates multiple pro-apoptotic signaling pathways, while mitochondrial calcium overload leads to mitochondrial membrane depolarization and cytochrome c release, thereby irreversibly initiating the apoptotic cascade.

In addition to the UPR, oxidative stress constitutes a second, reciprocally interacting stress axis in PMD pathogenesis. Disease-associated PLP1 mutations cause premature oligomerization of PLP in the ER, an event that correlates with disease severity and may disrupt ER-mitochondria communication at mitochondria-associated membranes (MAMs) [79]. In the PLP-tg 66/66 mouse model (PLP1 overexpression/duplication), Ruiz et al. documented oxidative damage to 21 proteins including glycolytic enzymes and OXPHOS components, decreased ATP, impaired mitochondrial dynamics (fission arrest), and a defective antioxidant response [176]. Patient-derived fibroblasts showed elevated mitochondrial ROS, membrane depolarization, and reduced respiratory capacity—all rescued by the antioxidant N-acetylcysteine [176].

Mechanistically, this reflects a vicious cycle: ER stress promotes ERO1-mediated H_2_O_2_ production during oxidative protein folding (amplified by CHOP-driven ERO1 upregulation); H_2_O_2_ activates IP3R-mediated ER Ca^2+^ release, which stimulates mitochondrial metabolism and further ROS production; mitochondrial ROS leaks back to the ER, aggravating protein misfolding and UPR activation [177]. Calnexin, which stably binds mutant PLP [166], localizes to MAMs and may couple ER stress to mitochondrial dysfunction [176]. Notably, this vicious cycle appears mutation-type dependent: previous work found little dysregulation of antioxidant pathways in missense-mutant mice (rsh, msd), suggesting UPR and oxidative stress are partially separable in these models [134], whereas the duplication/overexpression model shows clear oxidative stress [176]. This distinction has therapeutic implications—antioxidant strategies may be particularly relevant for PLP1 duplication PMD, while combination with UPR modulators may be required for missense-mutant forms.

These findings suggest that redox imbalance is not an isolated event but is tightly coupled to proteostasis failure, further amplifying ER stress and promoting OL apoptosis.

Therefore, we hypothesize that the primary function of UPR signaling is to alleviate the burden of misfolded proteins within the ER. Under PMD conditions, PERK-mediated upregulation of ERO1α in OLs may initially serve to enhance PDI oxidation, maintain optimal protein folding efficiency, and maintain ER redox homeostasis. However, persistent expression of mutant PLP appears to dysregulate ERO1α activity, resulting in excessive GSH consumption, increased H_2_O_2_ production, and sustained ER Ca^2+^ leakage, which collectively contribute to OL apoptosis. 

Currently, the majority of studies on ERO1α remain focused on the field of cancer, whereas systematic investigations in the nervous system have only begun to emerge in recent years [178]. The role of ERO1α in white matter disorders, including PMD, multiple sclerosis (MS), and Vanishing White Matter (VWM), may have been substantially underestimated, and ERO1α could represent an important driver of chronic neurodegeneration. Future investigations will aim to elucidate this mechanism.

## 6. Treatment and Prognosis of PMD

Connatal PMD, the most severe form, typically results in death by the second decade of life, whereas classical PMD is comparatively moderate, with survival extending into the third to seventh decade. PLP1 null mutations produce the mildest phenotype, characterized primarily by length-dependent axonal degeneration without significant dysmyelination. Most patients present with nystagmus, hypotonia progressing to spastic paraplegia, ataxia, and cognitive dysfunction; death often results from respiratory complications associated with motor impairment [179].

Current therapeutic strategies can be broadly grouped into three categories: (i) tolerance of abnormal or excessive PLP—enhancing mutant PLP folding, improving ER-to-membrane trafficking, or modulating the UPR to reduce proteotoxic stress; (ii) restoration of normal PLP levels—gene correction or splicing modulation to produce wild-type PLP at physiological levels; and (iii) reduction in abnormal or excessive PLP to a null-like state—suppressing mutant or overexpressed PLP1 via ASOs, RNAi, or CRISPR-Cas9, exploiting the observation that PLP1 loss-of-function phenotypes are milder than those caused by toxic gain-of-function mutations. Among these, PLP1 suppression is the strategy closest to clinical translation: Ionis Pharmaceuticals initiated a Phase 1b trial of the PLP1-targeting ASO ION356 (NCT06150716) in 2024, representing the first in-human trial of a PLP1 suppression therapeutic. Iron chelation with deferiprone and glial progenitor cell transplantation (Sana Biotechnologies’ SC379) are also advancing toward clinical evaluation [179].

Given that proteostasis collapse represents a central pathogenic mechanism in PMD, therapeutic strategies aimed at restoring ER homeostasis and reducing proteotoxic stress have emerged as promising approaches.

Currently, no highly targeted therapies have been successfully translated into clinical treatment for PMD patients. Nevertheless, based on current research progress, suppression of mutant protein production and modulation of the UPR signaling appear to be the most promising therapeutic strategies. Given the complex relationship between PMD genotypes and phenotypes, precision-targeted approaches are likely required to achieve optimal therapeutic efficacy [180].

Several potential therapeutic strategies have been investigated. Targeting intracellular signaling pathways represents one promising approach. Inhibition of the MEK/ERK pathway using compounds such as U0126 or PD98059 can ameliorate defects in OL differentiation and myelination induced by PLP1 overexpression [181]. Metabolic interventions have also shown therapeutic potential; notably, a high-cholesterol diet improved pathological manifestations and symptoms in PMD mouse models, supporting dietary modulation as a possible adjunctive therapy. Together, cell replacement strategies, including transplantation of neural stem cells or hematopoietic progenitor cells, have generated myelin-forming OLs in animal models and early-stage clinical studies, highlighting a promising regenerative approach [182,183]. Therapies targeting the UPR signaling have also shown promising effects, with several small molecules, including Salubrinal, Guanabenz, and Sephin1, improving myelin preservation, remyelination, and functional recovery in experimental models [179,184].

Suppression of mutant PLP1 expression has also emerged as a promising therapeutic strategy [179]. Reduction in toxic or excessive PLP1 expression through antisense oligonucleotides (ASOs), RNA interference (RNAi), or CRISPR-Cas9-mediated gene editing significantly prolonged survival, restored myelination, and improved motor function in multiple mouse models. Iron chelation using deferiprone demonstrated therapeutic benefits in selected cellular and animal studies. Furthermore, 4-phenylbutyric acid (4-PBA) improved cell viability and reduced ER stress-related markers at the cellular level [185]. Adeno-associated virus (AAV)-based therapies have also effectively suppressed mutant or duplicated PLP expression, providing important preclinical evidence supporting the feasibility of in vivo gene therapy for PMD [186].

Oral administration of curcumin significantly prolonged the lifespan of msd mice by approximately 25% and markedly reduced the number of apoptotic OLs within CNS white matter regions. However, curcumin treatment did not improve motor performance, neurological phenotypes, or myelination in these mice. Neither MBP expression nor myelin ultrastructure showed substantial recovery after treatment. It has been proposed that curcumin exerts its effects through combined anti-inflammatory and antioxidant activities, which may provide greater benefit in patients with milder disease phenotypes [187,188].

Piracetam, a nootropic agent whose primary pharmacological action is restoration of cell membrane fluidity through interaction with phospholipid polar headgroups, was identified through a drug screen as a lead compound capable of improving mutant PLP trafficking [189]. In MO3.13 OL-derived cells expressing the msd mutation, piracetam acted as a pharmacological chaperone: it significantly increased total mutant PLP1 protein levels, promoted its relocalization from the ER to the cell membrane, decreased ER stress markers, and reversed msd mutant-induced gene expression changes in microarray analysis [189]. These effects are consistent with piracetam’s known mechanism of stabilizing membrane structure, which facilitates proper folding and trafficking of transmembrane proteins. However, despite its beneficial effects in vitro, piracetam treatment failed to significantly prolong survival or improve body weight gain in msd mice, suggesting that partial chaperone rescue of mutant PLP trafficking is insufficient to overcome the proteotoxic burden in vivo [189]. It is necessary to combine this agent with other treatment methods to better exert its effects. 

Similarly, chloroquine (CQ) reduces ER stress in PMD through a distinct mechanism: rather than improving protein folding, CQ enhances phosphorylation of eukaryotic initiation factor 2 alpha (eIF2α), thereby attenuating global cap-dependent translation and reducing the influx of newly synthesized misfolded PLP1 into the ER [190]. In HeLa cells expressing msd mutant, CQ decreased the amount of ER-resident mutant protein and ameliorated ER stress. CQ also attenuated ER stress in primary oligodendrocytes from msd mice. In vivo, CQ crossed the blood–brain barrier, inhibited ER stress markers in the spinal cord, and upregulated mature OL marker gene expression [190]. Notably, CQ’s efficacy was specific to ER stress induced by misfolded nascent proteins: it attenuated stress caused by Tm but not by Tg, confirming that its mechanism targets the burden of unfolded nascent proteins entering the ER—the precise pathogenic mechanism in missense-mutation PMD [190]. CQ thus belongs to the same mechanistic class as guanabenz and salubrinal, which also increase eIF2α phosphorylation to reduce proteotoxic load [179]. Nevertheless, although CQ attenuated ER stress and promoted OL differentiation markers at the cellular level, it did not fully prevent apoptosis or significantly extend the lifespan of msd mice, indicating that translational attenuation alone is insufficient to rescue the disease [190]. It is also necessary to combine with other treatment methods to better exert its effects, and it is necessary to prescribe the right treatment based on the specific condition.

Collectively, these therapeutic approaches converge on a common goal of restoring proteostasis, further supporting the concept that PMD is fundamentally a proteostasis-driven disorder.

## 7. Conclusions

PLP1-associated leukodystrophies represent a group of genetically and clinically heterogeneous myelin disorders in which oligodendrocytes are particularly vulnerable to disturbances in ER proteostasis. Accumulating evidence indicates that mutant PLP1 proteins induce chronic ER stress, disrupt protein quality control systems, and trigger maladaptive unfolded protein response signaling, ultimately leading to OL dysfunction and myelin degeneration. Recent advances have substantially improved our understanding of the molecular mechanisms linking PLP1 mutations to oligodendroglial pathology, particularly the roles of ER chaperones, ER-associated degradation, and UPR pathways. Nevertheless, the precise determinants underlying phenotypic heterogeneity and selective OL vulnerability remain incompletely understood. Future studies integrating molecular, cellular, and translational approaches will be essential for identifying effective targeted therapies and advancing precision medicine for PLP1-associated leukodystrophies.

## Data Availability

No new data were created or analyzed in this study. Data sharing is not applicable to this article.

## Abbreviations

The following abbreviations are used in this manuscript:

ABR	Auditory Brainstem Response
AIF	Apoptosis-Inducing Factor
ASO	Antisense oligonucleotide
ATF3	Activating Transcription Factor 3
ATF4	Activating Transcription Factor 4
ATF6	Activating Transcription Factor 6
Bax	BCL2-associated X protein
Bak	BCL2 antagonist/killer
BiP	Binding immunoglobulin protein (also known as GRP78)
Bim	BCL2-interacting mediator of cell death
CALR	Calreticulin
CHOP	C/EBP homologous protein
CNS	Central nervous system
CNX	Calnexin
COPI	Coat protein complex I
COPII	Coat protein complex II
CST	Cerebroside sulfotransferase
DM20	DM20 isoform of proteolipid protein 1
EC1	Extracellular loop 1
EC2	Extracellular loop 2
EDEM	ER degradation-enhancing α-mannosidase-like protein
eIF2α	Eukaryotic translation initiation factor 2 alpha
ER	Endoplasmic reticulum
ERAD	Endoplasmic reticulum-associated degradation
ERES	Endoplasmic reticulum exit site
ERO1α	Endoplasmic reticulum oxidoreductin 1 alpha
ERQC	Endoplasmic reticulum quality control
ERS	Endoplasmic reticulum stress
ESS	Exonic splicing silencer
GA	Golgi apparatus
GalC	Galactosylceramide
GADD34	Growth arrest and DNA damage-inducible protein 34
GFAP	Glial fibrillary acidic protein
GRP78	Glucose-regulated protein 78
GSH	Reduced glutathione
GSSG	Oxidized glutathione
HEMS	Hypomyelination of early myelinating structures
HLD	Hereditary leukodystrophy
IBA1	Ionized calcium-binding adapter molecule 1
IICR	IP3-induced calcium release
IPL	Intraperiod line
IP3R	Inositol 1,4,5-trisphosphate receptor
IRE1	Inositol-requiring enzyme 1
ISE	Intronic splicing enhancer
JNK	c-Jun N-terminal kinase
KDEL	Lys-Asp-Glu-Leu endoplasmic reticulum retrieval motif
LE/Lys	Late endosome/lysosome
MAG	Myelin-associated glycoprotein
MAL	Myelin and lymphocyte protein
MAM	Mitochondria-associated membrane
MBP	Myelin basic protein
MDL	Major dense line
MHC II	Major histocompatibility complex class II
MOG	Myelin oligodendrocyte glycoprotein
MRI	Magnetic resonance imaging
MRI-T2WI	T2-weighted magnetic resonance imaging
NE	Nuclear envelope
NLD	N-terminal luminal domain
NMD	Nonsense-mediated mRNA decay
OL	Oligodendrocyte
OPC	Oligodendrocyte precursor cell
OSP	Oligodendrocyte-specific protein (Claudin-11)
PARP	Poly(ADP-ribose) polymerase
PDI	Protein disulfide isomerase
PERK	PKR-like endoplasmic reticulum kinase
PLP	Proteolipid protein
PLP1	Proteolipid protein 1
PMD	Pelizaeus–Merzbacher disease
PTC	Premature termination codon
QKI	Quaking
RE	Recycling endosome
RIDD	Regulated IRE1-dependent decay
RNase	Ribonuclease
ROS	Reactive oxygen species
SCI	Spinal cord injury
SDS	Sodium dodecyl sulfate
SERCA	Sarco/endoplasmic reticulum Ca^2+^-ATPase
SIRT2	Sirtuin 2
SNARE	Soluble NSF attachment protein receptor
SPG2	Spastic paraplegia type 2
TGN	Trans-Golgi network
TMD	Transmembrane domain
TRAF2	TNF receptor-associated factor 2
TRIB3	Tribbles pseudokinase 3
UPR	Unfolded protein response
UPRE	Unfolded protein response element
UPS	Ubiquitin–proteasome system
VAMP	Vesicle-associated membrane protein
VEP	Visual evoked potential
XBP1	X-box binding protein 1
